# Supplementary material for: Efficient Induction of Antigen-Specific CD8+ T-Cell Responses by Cationic Peptide-Based mRNA Nanoparticles
Source: Pharmaceutics. 2022 Jun 30;14(7):1387. doi: 10.3390/pharmaceutics14071387 (PMC9321026; doi:10.3390/pharmaceutics14071387)
Supplement: Supplementary file 1 [file pharmaceutics-14-01387-s001.zip › pharmaceutics-1789395-supplementary.pdf]

pharmaceutics

Supplementary Materials: Efficient Induction of Antigen-Specific CD8+ T-Cell Responses by Cationic-Peptide-Based mRNA Nanoparticles

Sigrid D'haese, Thessa Laeremans, Sabine den Roover, Sabine D. Allard, Guido Vanham and Joeri L. Aerts

Table S1. Antibodies used for flow cytometry analysis.

Target	Clone	Fluorophore
CD11c	N418	Pe-Cy7
B220	RA3-6B2	APC-Cy7
Clec9a	7H11	PE
CD24	30-F1	APC
CD103	2E7	PerCP-Cy5.5
MHCI-S	25-D1.16	PE
CD40	3/23	APC
CD80	16-10A1	BV421
CD86	GL-1	PE-CF594
CD45.1	A20	PE
CD45.2	104	Spark NIR™ 685
CD3e	145-2C11	FITC
CD8	53-6.7	BV510
IFN-γ	XMG1.2	AF700
TNF-α	MP6-XT22	PE-Cy7
IL-2	JES6 5H4	APC

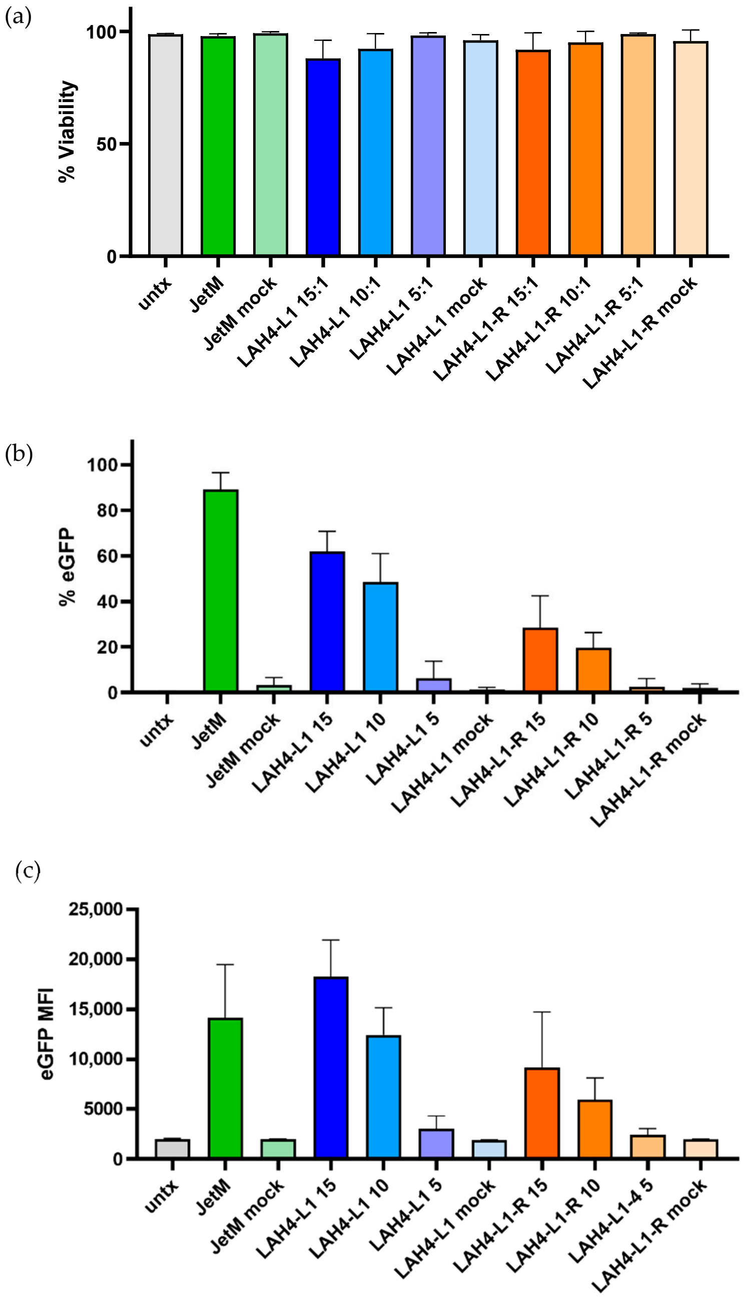

Figure S1. Transfection of histidine rich peptides-mRNA nanoparticles (NPs) in DC2.4 cells. DC2.4 were transfected with 250 ng mRNA encoding eGFP per 200,000 cells at indicated ratios mRNA:HPR (µg:µg, 15 indicating 1:15 etc.), the next day cells were harvested and read out was performed by flow cytometry. As a negative control, DC2.4 cells were left untreated (untx) or mock treated (no mRNA). As a positive control, DC2.4 cells were transfected with jetMessenger® (JetM). (**a**) Viability, (**b**) eGFP percentage (**c**) eGFP MFI after transfection. (n = 4, mean ± SD).

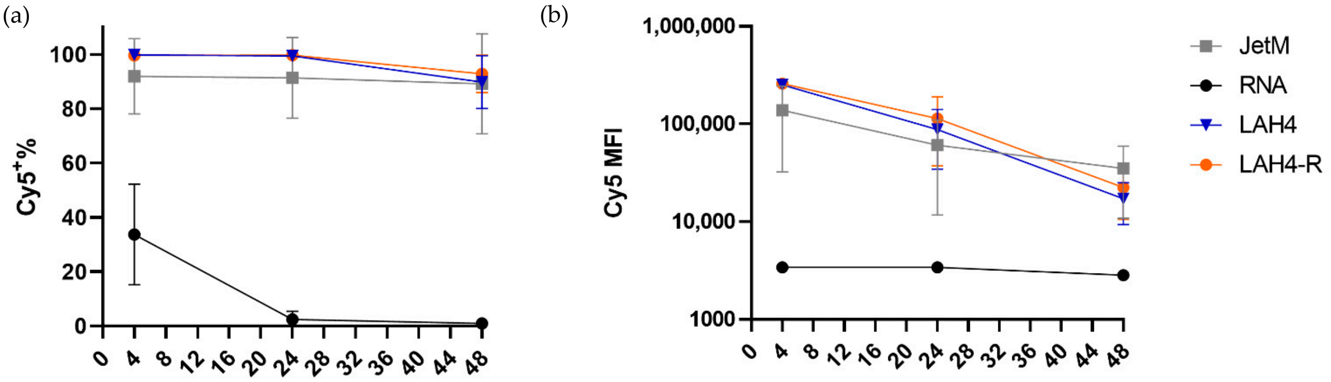

Figure S2. Histidine rich peptides allow efficient uptake in DC2.4 cells. DC2.4 cells were transfected at a ratio 1:15 (µg mRNA:µg HRP) with 125 ng Cy5 labelled mRNA encoding eGFP for 100,000 cells. Mock conditions (without mRNA), and untreated conditions not shown on the graph. Read out was performed after 4, 24 and 48 hours by flow cytometry for detection of percentage Cy5⁺ cells (**a**) and MFI of Cy5 (**b**) (n = 3, mean ± SD).

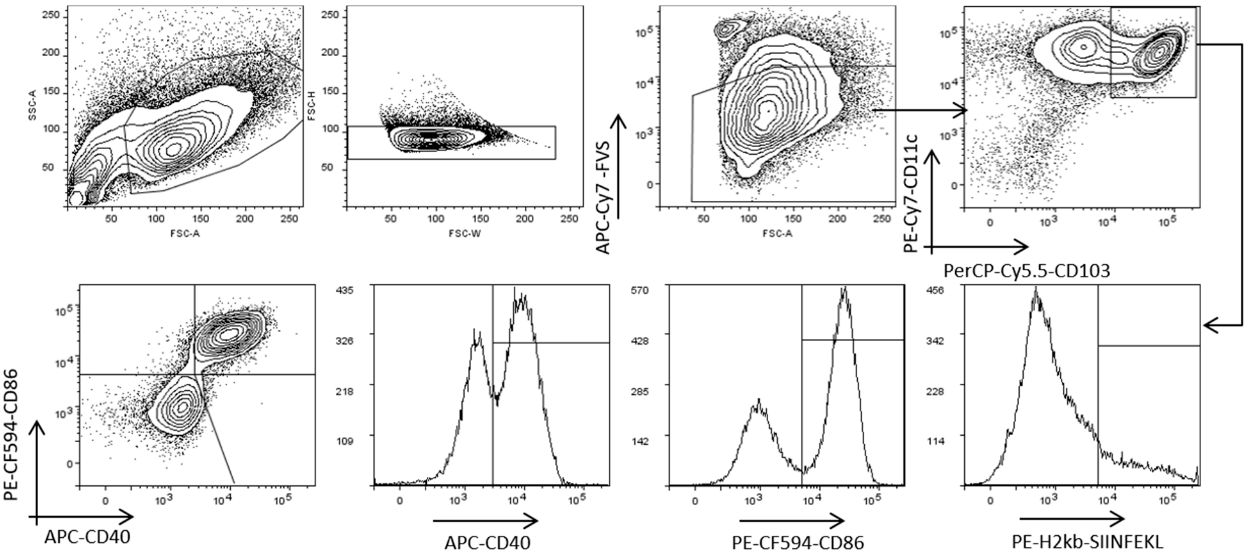

Figure S3. Gating strategy for bone marrow derived CD103⁺ DCs. CD103⁺ DCs were generated and transfected on day 13 with mRNA encoding tOVA (unmodified, RNA/modified, MOD). The next day cells were harvested and stained for, viability (with fixable viability staining or FVS), DC markers (CD103 and CD11c), maturation markers (CD86 and CD40) and SIINFEKL presented in MHC I (H2kb-SIINFEKL).

(a)

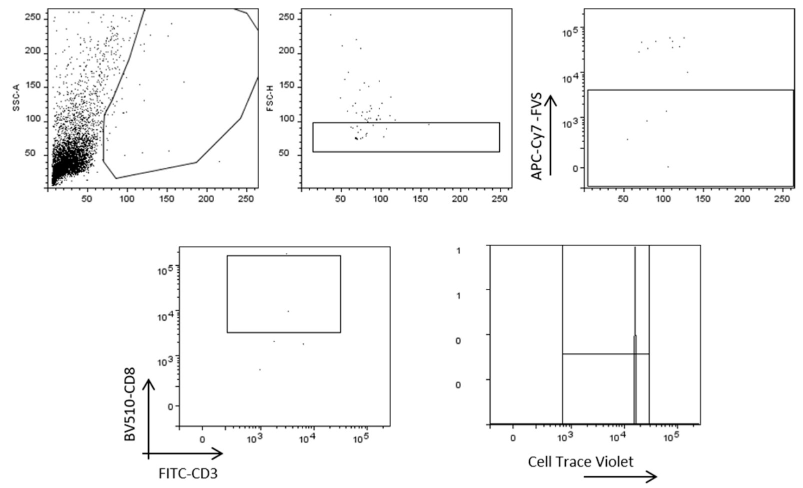

(b)

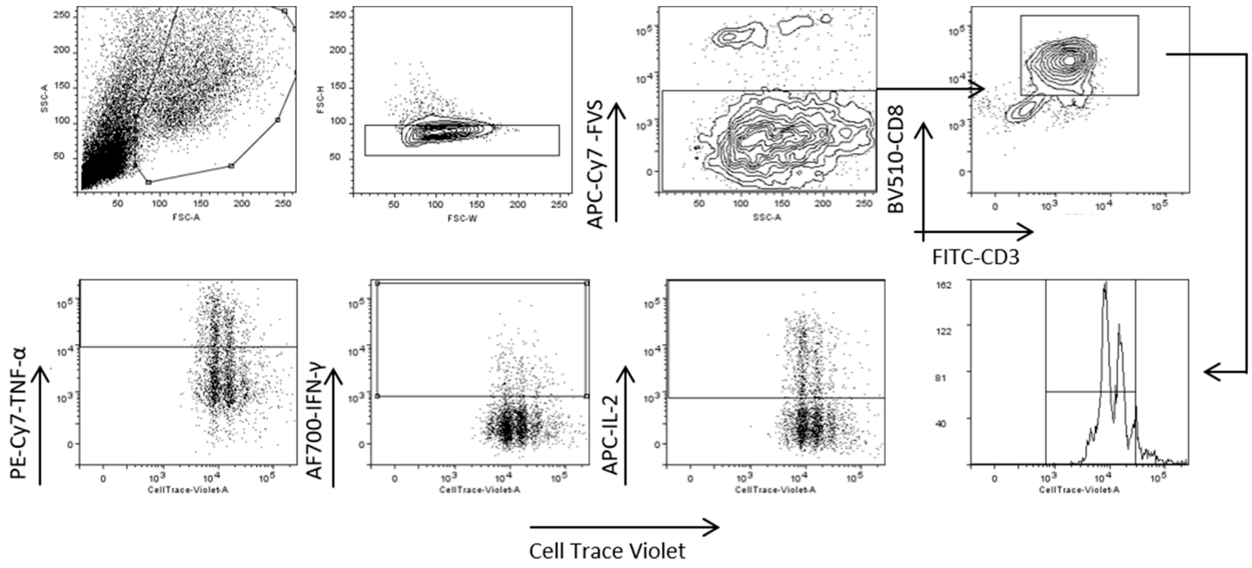

Figure S4. Gating strategy for polyfunctionality of OT-I splenocytes. Transfected $CD103^+$ DCs were co-cultured with OT-I splenocytes for 3 days. On the second day, monensin and brefeldin A were added overnight to block cytokine secretion. The next day, polyfunctionality was assessed within the $CD3^+$ $CD8^+$ T cell population. The 4 functions assessed were proliferation (dilution of CTV, ≥ 3 divisions) and TNF-α, IFN-γ and IL-2 production. (**a**) Gating strategy for the negative control LAH4-L1 mock. (**b**) Gating strategy for OT-I cells co-cultured with $CD103^+$ DCs tranfsected with LAH4-L1 mRNA nanoparticles.

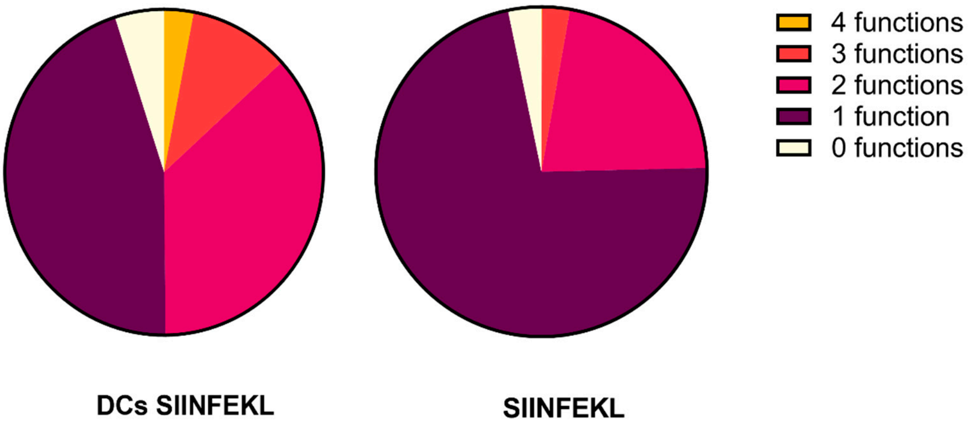

Figure S5. Pie charts for polyfunctionality in OT-I splenocytes stimulated with SIINFEKL or co-cultured with CD103⁺ DCs pulsed with SIINFEKL (DCs SIINFEKL). OT-I splenocytes were culture with SIINFEKL or CD103⁺ DCs pulsed with SIINFEKL for 3 days. On the second day, monensin and brefeldin A were added overnight to block cytokine secretion. The next day, polyfunctionality was assessed within the CD3⁺ CD8⁺ T cell population. The 4 functions assessed were proliferation (dilution of CTV) and TNF-α, IFN-γ and IL-2 production.
